# Boc-AzAla-Ala-OMe

**DOI:** 10.1107/S1600536809045498

**Published:** 2009-11-14

**Authors:** Cécile Abbas, Brigitte Jamart Grégoire, Régis Vanderesse, Claude Didierjean

**Affiliations:** a Laboratoire de Chimie Physique Macromoléculaire, UMR CRNS-INPL 7568, Nancy Université, BP 451, 54001 Nancy, France; b Laboratoire de Cristallographie, Résonance Magnétique et Modélisations (CRM^2^), Nancy Université, UMR CNRS-UHP 7036, BP 70236, 54506 Vandoeuvre-lès-Nancy, France

## Abstract

The title compound (systematic name: *tert*-butyl 3-{[1-(methoxy­carbon­yl)eth­yl]amino­carbon­yl}-3-methyl­carbazate), C_11_H_21_N_3_O_5_, is a precursor for the study of a new class of foldamer based on aza/α-dipeptide oligomerization [Abbas *et al.* (2009 ▶). *Tetra­hedron Lett.* **50**, 4158–4160]. The asymmetric unit consists of one mol­ecule in an extended conformation which is stabilized by inter­molecular N—H⋯O and C—H⋯O hydrogen bonding.

## Related literature

For the synthesis, see: Majer & Randad (1994 ▶); Brosse *et al.* (2001 ▶); Bouillon *et al.* (2004 ▶); Abbas *et al.* (2009 ▶). For the geometry of the aza-residue in aza­peptides, see: Benatalah *et al.* (1991 ▶), André *et al.* (1996 ▶). For Boc-AzAla-Pro-NHiPr, see: André *et al.* (1997 ▶). For the refinement procedure, see: Flack & Schwarzenbach (1988 ▶). For hydrogen-bond motifs, see: Etter (1990 ▶).
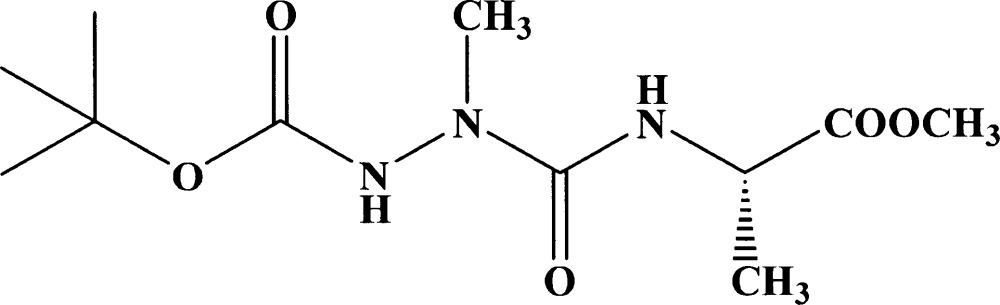


## Experimental

### 

#### Crystal data

C_11_H_21_N_3_O_5_
*M* *_r_* = 275.31
Tetragonal, 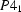

*a* = 9.3194 (4) Å
*c* = 17.4420 (8) Å
*V* = 1514.86 (12) Å^3^
*Z* = 4
Mo *K*α radiation
μ = 0.10 mm^−1^
*T* = 100 K
0.3 × 0.2 × 0.2 mm

#### Data collection

Nonius KappaCCD diffractometer
Absorption correction: none
14534 measured reflections
1847 independent reflections
1806 reflections with *I* > 2σ(*I*)
*R*_int_ = 0.049

#### Refinement

*R*[*F* ^2^ > 2σ(*F* ^2^)] = 0.036
*wR*(*F* ^2^) = 0.076
*S* = 1.12
1847 reflections
178 parameters
1 restraint
H-atom parameters constrained
Δρ_max_ = 0.17 e Å^−3^
Δρ_min_ = −0.13 e Å^−3^

### 

Data collection: *COLLECT* (Bruker, 2004 ▶); cell refinement: *SCALEPACK* (Otwinowski & Minor, 1997 ▶); data reduction: *DENZO* (Otwinowski & Minor, 1997 ▶) and *SCALEPACK*; program(s) used to solve structure: *SIR92* (Altomare *et al.*, 1994 ▶); program(s) used to refine structure: *SHELXL97* (Sheldrick, 2008 ▶); molecular graphics: *ORTEP-3 for Windows* (Farrugia, 1997 ▶) and *PLATON* (Spek, 2009 ▶); software used to prepare material for publication: *WinGX* (Farrugia, 1999 ▶).

## Supplementary Material

Crystal structure: contains datablocks global, I. DOI: 10.1107/S1600536809045498/dn2501sup1.cif

Structure factors: contains datablocks I. DOI: 10.1107/S1600536809045498/dn2501Isup2.hkl

Additional supplementary materials:  crystallographic information; 3D view; checkCIF report

## Figures and Tables

**Table 1:** Hydrogen-bond geometry (Å, °)

*D*—H⋯*A*	*D*—H	H⋯*A*	*D*⋯*A*	*D*—H⋯*A*
N1—H1⋯O2^i^	0.86	2.18	2.972 (2)	153
N3—H3⋯O3^i^	0.86	2.03	2.850 (2)	159
C6—H6*A*⋯O4^ii^	0.96	2.55	3.303 (3)	136
C11—H11*B*⋯O4^iii^	0.96	2.41	3.346 (3)	164

Symmetry codes: (i) 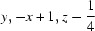; (ii) 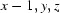; (iii) 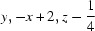.

**Fig. 1.**
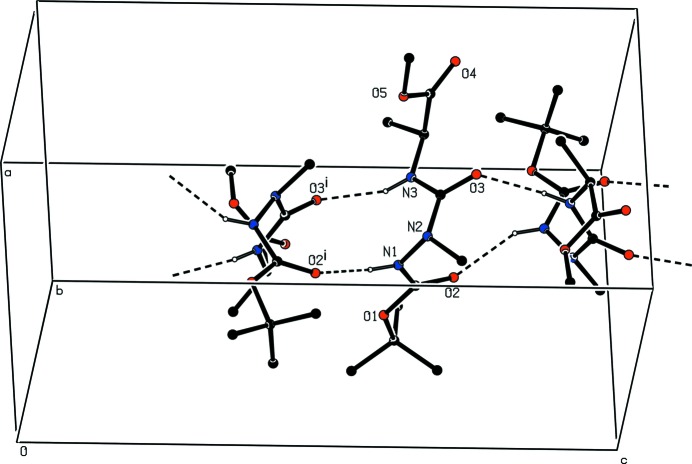
The molecular structure of the title compound showing the atom-numbering scheme. All non-H atoms are represented by 25% probability displacement ellipsoids.

**Fig. 2.**
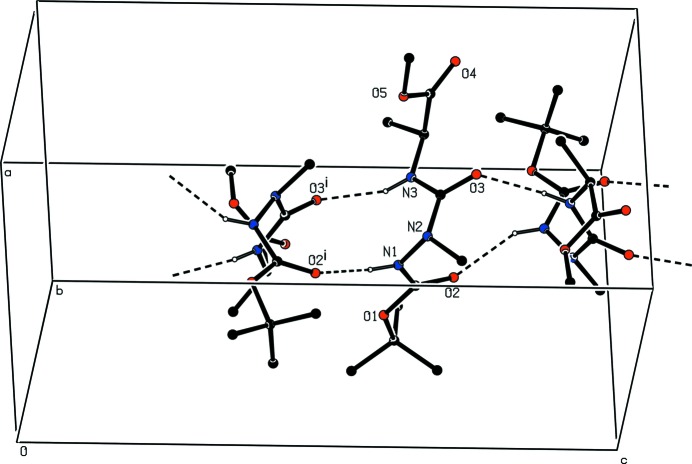
Partial packing view of the title compound showing the formation of the C(4)C(4)[R22(12)] graph set motif paralell to [001]. H atoms not involved in hydrogen bondings have been omitted for clarity. H bonds are shown as dashed lines. [Symmetry code: (i) y, -x+1, z-1/4]

## supplementary crystallographic information

### Comment

As part of our continuing studies on the synthesis and structure of hydrazino-and *N*-amino-peptides, we recently described the original and efficient synthesis of aza/α-dipeptides *via* Mitsunobu and *trans*-protections protocols starting from *N*-*tert*-butyloxycarbonylaminophtalimide (Abbas *et al.*, 2009; Majer & Randad 1994; Brosse *et al.* 2001; Bouillon *et al.* 2004). Aza-peptides are pseudopeptides, in which nitrogen has been substituted for at least one of the CH^α^ groups. Here we report the crystal structure of the pseudodipeptide Boc-AzAla-Ala-OMe (Fig. 1).

In the present study, the geometry of the aza-residue is similar to those observed for known azapeptides structures. In most of the cases, the α-nitrogen adopts a non-planar planar structure (André *et al.*, 1996; Benatalah *et al.* 1991). In the title compound, the deviation of the α-nitrogen out of the plane, defined by the three atoms bonded to it, is 0.268 (2) Å. The greatest difference from standard peptide group concern the bond lengths and bond angles around the α-nitrogen: (i) the N—N^α^ (N1—N2) and N^α^—C^β^ (N2—C6) bonds are shorter by about 0.06Å than their homologous bonds in peptides; (ii) the N^α^—C' ((N2—C7) bond of 1.392 (3)Å is shorter than the homologous C^α^—C' bond and exceeds the dimension of the amide bond; (iii) the bond angles around the α-nitrogen are larger by 5–6° than the bond angles around the α-carbon.

In the solid state, Boc-AzAla-Pro-NHiPr (André *et al.*, 1997) and the title compound Boc-AzAla-Ala-OMe adopt two distinct conformations with the N^α^ atoms having opposite configurations. In the former, the AzAla residue assumes the *R* chirality and the pseudodipeptide is folded by an intramolecular hydrogen bond between the (iPr)NH and the Boc(CO) groups. In the crystal of the title compound, the pseudodipeptides adopt an extended conformation which form infinite chains along the four fold axis. The N-H goups of AzaAla and Ala residues are engaged in intermolecular hydrogen bonds with the carbonyl groups of the N-terminal protecting group and the aza-residue, respectively, forming two C(4) chain motifs (Fig. 2, Etter 1990). Combination of these two motifs generates a new R^2^_2_(12) pattern, shown in the Fig. 2. Finally, the third carbonyl group, C10=O4, is involved in weak CH···O hydrogen bonds forming a threedimensional network structure.

### Experimental

The title compound was prepared from *N*-*tert*-butyloxycarbonylaminophtalimide (Abbas *et al.*, 2009), and was crystallized by slow evaporation of a diethyl ether solution.

### Refinement

Because of the lack of any significant anomalous dispersion effects, the absolute configurations of the title compound could not be determined from the diffraction experiments but was known from the method of synthesis. The origin was fixed by floating-origin restraints (Flack & Schwarzenbach, 1988). All H atoms were located in difference Fourier maps. The C/N-bonded H atoms were placed at calculated positions and refined using a riding model, with C—H distances of 0.96–0.98 Å and with N—H distance of 0.86 Å. The H-atom *U*_iso_ parameters were fixed at 1.2Ueq(C) for methine C—H, at 1.2Ueq(N) for the N—H group and at 1.5Ueq(C) for methyl C—H.

### Crystal data

**Table:**

C_11_H_21_N_3_O_5_	*D*_x_ = 1.207 Mg m^−^^3^
*M**_r_* = 275.31	Mo *K*α radiation, λ = 0.71073 Å
Tetragonal, *P*4_1_	Cell parameters from 14534 reflections
Hall symbol: P 4w	θ = 2.5–27.9°
*a* = 9.3194 (4) Å	µ = 0.10 mm^−^^1^
*c* = 17.4420 (8) Å	*T* = 100 K
*V* = 1514.86 (12) Å^3^	Prism, colorless
*Z* = 4	0.3 × 0.2 × 0.2 mm
*F*(000) = 592	

### Data collection

**Table:**

Nonis KappaCCD diffractometer	*R*_int_ = 0.049
CCD rotation images, thick slices scans	θ_max_ = 27.8°, θ_min_ = 2.5°
14534 measured reflections	*h* = −12→12
1847 independent reflections	*k* = −12→12
1806 reflections with *I* > 2σ(*I*)	*l* = −22→22

### Refinement

**Table:**

Refinement on *F*^2^	1 restraint
Least-squares matrix: full	H-atom parameters constrained
*R*[*F*^2^ > 2σ(*F*^2^)] = 0.036	*w* = 1/[σ^2^(*F*_o_^2^) + (0.0164*P*)^2^ + 0.7016*P*] where *P* = (*F*_o_^2^ + 2*F*_c_^2^)/3
*wR*(*F*^2^) = 0.076	(Δ/σ)_max_ < 0.001
*S* = 1.12	Δρ_max_ = 0.17 e Å^−^^3^
1847 reflections	Δρ_min_ = −0.13 e Å^−^^3^
178 parameters	

### Special details

**Table:**

Geometry. All e.s.d.'s (except the e.s.d. in the dihedral angle between two l.s. planes) are estimated using the full covariance matrix. The cell e.s.d.'s are taken into account individually in the estimation of e.s.d.'s in distances, angles and torsion angles; correlations between e.s.d.'s in cell parameters are only used when they are defined by crystal symmetry. An approximate (isotropic) treatment of cell e.s.d.'s is used for estimating e.s.d.'s involving l.s. planes.

### Fractional atomic coordinates and isotropic or equivalent isotropic displacement parameters (Å^2^)

**Table:**

	*x*	*y*	*z*	*U*_iso_*/*U*_eq_	
C1	0.3488 (2)	0.0563 (2)	0.63461 (13)	0.0228 (4)	
C2	0.2822 (3)	−0.0204 (3)	0.56621 (14)	0.0311 (5)	
H2A	0.1902	0.021	0.5551	0.047*	
H2B	0.2706	−0.1204	0.578	0.047*	
H2C	0.3438	−0.0103	0.5224	0.047*	
C3	0.2529 (3)	0.0415 (2)	0.70463 (14)	0.0279 (5)	
H3A	0.2995	0.0837	0.7482	0.042*	
H3B	0.235	−0.0582	0.7145	0.042*	
H3C	0.1635	0.0898	0.6954	0.042*	
C4	0.5016 (3)	0.0058 (3)	0.64752 (15)	0.0308 (5)	
H4A	0.557	0.022	0.6019	0.046*	
H4B	0.5015	−0.0948	0.6594	0.046*	
H4C	0.5431	0.0583	0.6894	0.046*	
O1	0.35134 (16)	0.20833 (15)	0.60837 (9)	0.0218 (3)	
C5	0.3987 (2)	0.3109 (2)	0.65626 (12)	0.0193 (4)	
O2	0.43957 (17)	0.29435 (17)	0.72181 (9)	0.0239 (3)	
N1	0.39666 (19)	0.44041 (18)	0.61993 (11)	0.0201 (4)	
H1	0.3753	0.4464	0.5721	0.024*	
N2	0.43006 (19)	0.56280 (19)	0.66214 (11)	0.0200 (4)	
C6	0.3156 (2)	0.6117 (2)	0.71325 (14)	0.0259 (5)	
H6A	0.2304	0.63	0.6839	0.039*	
H6B	0.3451	0.6982	0.7386	0.039*	
H6C	0.2961	0.5389	0.7508	0.039*	
C7	0.5716 (2)	0.5760 (2)	0.68660 (12)	0.0189 (4)	
O3	0.60321 (16)	0.65021 (16)	0.74280 (8)	0.0221 (3)	
N3	0.67193 (19)	0.50835 (19)	0.64451 (10)	0.0209 (4)	
H3	0.6487	0.4606	0.6042	0.025*	
C8	0.8201 (2)	0.5181 (2)	0.66902 (13)	0.0232 (4)	
H8	0.8264	0.478	0.7209	0.028*	
C9	0.9146 (2)	0.4274 (3)	0.61671 (14)	0.0296 (5)	
H9A	0.8853	0.3288	0.6198	0.044*	
H9B	1.0129	0.4358	0.6326	0.044*	
H9C	0.9052	0.4605	0.5648	0.044*	
C10	0.8773 (2)	0.6709 (3)	0.67187 (13)	0.0244 (4)	
O4	0.97394 (18)	0.7060 (2)	0.71417 (11)	0.0333 (4)	
O5	0.81613 (16)	0.75824 (17)	0.62089 (9)	0.0249 (3)	
C11	0.8685 (3)	0.9048 (3)	0.62253 (16)	0.0310 (5)	
H11A	0.8424	0.9487	0.6703	0.046*	
H11B	0.8267	0.9578	0.581	0.046*	
H11C	0.971	0.9048	0.6174	0.046*	

### Atomic displacement parameters (Å^2^)

**Table:**

	*U*^11^	*U*^22^	*U*^33^	*U*^12^	*U*^13^	*U*^23^
C1	0.0313 (11)	0.0171 (9)	0.0199 (10)	−0.0034 (8)	0.0039 (9)	0.0017 (8)
C2	0.0429 (14)	0.0269 (12)	0.0236 (11)	−0.0138 (10)	0.0058 (10)	−0.0039 (9)
C3	0.0345 (12)	0.0232 (10)	0.0261 (11)	−0.0033 (9)	0.0091 (10)	−0.0012 (9)
C4	0.0339 (11)	0.0269 (11)	0.0317 (12)	0.0044 (9)	0.0059 (10)	0.0039 (10)
O1	0.0288 (8)	0.0190 (7)	0.0178 (7)	−0.0036 (6)	−0.0026 (6)	0.0000 (6)
C5	0.0183 (9)	0.0217 (10)	0.0180 (10)	−0.0021 (7)	0.0011 (8)	−0.0017 (8)
O2	0.0304 (8)	0.0240 (8)	0.0172 (7)	−0.0031 (6)	−0.0033 (6)	0.0007 (6)
N1	0.0251 (9)	0.0199 (8)	0.0155 (8)	−0.0020 (7)	−0.0033 (7)	−0.0001 (7)
N2	0.0217 (8)	0.0204 (8)	0.0178 (8)	−0.0010 (6)	0.0005 (7)	−0.0014 (7)
C6	0.0224 (10)	0.0256 (11)	0.0295 (12)	0.0017 (8)	0.0029 (9)	−0.0037 (9)
C7	0.0213 (10)	0.0190 (9)	0.0164 (10)	−0.0028 (8)	0.0009 (7)	0.0023 (8)
O3	0.0240 (8)	0.0256 (8)	0.0165 (7)	−0.0023 (6)	0.0026 (6)	−0.0024 (6)
N3	0.0197 (8)	0.0273 (9)	0.0157 (8)	−0.0014 (7)	0.0003 (7)	−0.0035 (7)
C8	0.0212 (10)	0.0306 (11)	0.0179 (10)	0.0013 (8)	−0.0020 (8)	0.0005 (9)
C9	0.0231 (11)	0.0413 (13)	0.0243 (11)	0.0049 (9)	0.0008 (9)	−0.0043 (10)
C10	0.0186 (10)	0.0357 (12)	0.0188 (10)	0.0012 (9)	0.0007 (8)	−0.0009 (9)
O4	0.0263 (8)	0.0421 (10)	0.0316 (9)	−0.0027 (7)	−0.0105 (7)	−0.0012 (8)
O5	0.0228 (8)	0.0301 (8)	0.0217 (8)	−0.0039 (6)	−0.0038 (6)	0.0018 (7)
C11	0.0283 (12)	0.0330 (12)	0.0317 (12)	−0.0052 (9)	−0.0019 (10)	0.0031 (10)

### Geometric parameters (Å, °)

**Table:**

C1—O1	1.489 (2)	C6—H6A	0.96
C1—C4	1.517 (3)	C6—H6B	0.96
C1—C3	1.520 (3)	C6—H6C	0.96
C1—C2	1.523 (3)	C7—O3	1.235 (3)
C2—H2A	0.96	C7—N3	1.346 (3)
C2—H2B	0.96	N3—C8	1.448 (3)
C2—H2C	0.96	N3—H3	0.86
C3—H3A	0.96	C8—C10	1.521 (3)
C3—H3B	0.96	C8—C9	1.524 (3)
C3—H3C	0.96	C8—H8	0.98
C4—H4A	0.96	C9—H9A	0.96
C4—H4B	0.96	C9—H9B	0.96
C4—H4C	0.96	C9—H9C	0.96
O1—C5	1.344 (2)	C10—O4	1.210 (3)
C5—O2	1.215 (3)	C10—O5	1.334 (3)
C5—N1	1.363 (3)	O5—C11	1.450 (3)
N1—N2	1.393 (2)	C11—H11A	0.96
N1—H1	0.86	C11—H11B	0.96
N2—C7	1.392 (3)	C11—H11C	0.96
N2—C6	1.463 (3)		
			
O1—C1—C4	109.01 (17)	N2—C6—H6A	109.5
O1—C1—C3	110.01 (17)	N2—C6—H6B	109.5
C4—C1—C3	113.9 (2)	H6A—C6—H6B	109.5
O1—C1—C2	102.26 (17)	N2—C6—H6C	109.5
C4—C1—C2	110.7 (2)	H6A—C6—H6C	109.5
C3—C1—C2	110.32 (19)	H6B—C6—H6C	109.5
C1—C2—H2A	109.5	O3—C7—N3	122.0 (2)
C1—C2—H2B	109.5	O3—C7—N2	121.24 (19)
H2A—C2—H2B	109.5	N3—C7—N2	116.72 (18)
C1—C2—H2C	109.5	C7—N3—C8	118.14 (18)
H2A—C2—H2C	109.5	C7—N3—H3	120.9
H2B—C2—H2C	109.5	C8—N3—H3	120.9
C1—C3—H3A	109.5	N3—C8—C10	113.75 (18)
C1—C3—H3B	109.5	N3—C8—C9	109.84 (18)
H3A—C3—H3B	109.5	C10—C8—C9	109.64 (19)
C1—C3—H3C	109.5	N3—C8—H8	107.8
H3A—C3—H3C	109.5	C10—C8—H8	107.8
H3B—C3—H3C	109.5	C9—C8—H8	107.8
C1—C4—H4A	109.5	C8—C9—H9A	109.5
C1—C4—H4B	109.5	C8—C9—H9B	109.5
H4A—C4—H4B	109.5	H9A—C9—H9B	109.5
C1—C4—H4C	109.5	C8—C9—H9C	109.5
H4A—C4—H4C	109.5	H9A—C9—H9C	109.5
H4B—C4—H4C	109.5	H9B—C9—H9C	109.5
C5—O1—C1	119.40 (17)	O4—C10—O5	124.0 (2)
O2—C5—O1	126.7 (2)	O4—C10—C8	122.3 (2)
O2—C5—N1	123.66 (19)	O5—C10—C8	113.58 (18)
O1—C5—N1	109.64 (18)	C10—O5—C11	114.71 (18)
C5—N1—N2	118.43 (18)	O5—C11—H11A	109.5
C5—N1—H1	120.8	O5—C11—H11B	109.5
N2—N1—H1	120.8	H11A—C11—H11B	109.5
C7—N2—N1	116.51 (17)	O5—C11—H11C	109.5
C7—N2—C6	118.49 (18)	H11A—C11—H11C	109.5
N1—N2—C6	114.46 (17)	H11B—C11—H11C	109.5
			
C4—C1—O1—C5	−65.6 (2)	C6—N2—C7—N3	168.95 (18)
C3—C1—O1—C5	60.0 (2)	O3—C7—N3—C8	3.2 (3)
C2—C1—O1—C5	177.16 (18)	N2—C7—N3—C8	−179.09 (18)
C1—O1—C5—O2	−1.3 (3)	C7—N3—C8—C10	−60.0 (3)
C1—O1—C5—N1	177.86 (17)	C7—N3—C8—C9	176.65 (19)
O2—C5—N1—N2	−6.4 (3)	N3—C8—C10—O4	153.4 (2)
O1—C5—N1—N2	174.45 (17)	C9—C8—C10—O4	−83.2 (3)
C5—N1—N2—C7	68.4 (2)	N3—C8—C10—O5	−30.0 (3)
C5—N1—N2—C6	−75.9 (2)	C9—C8—C10—O5	93.5 (2)
N1—N2—C7—O3	−156.14 (19)	O4—C10—O5—C11	−3.6 (3)
C6—N2—C7—O3	−13.3 (3)	C8—C10—O5—C11	179.84 (19)
N1—N2—C7—N3	26.1 (3)		

### Hydrogen-bond geometry (Å, °)

**Table:**

*D*—H···*A*	*D*—H	H···*A*	*D*···*A*	*D*—H···*A*
N1—H1···O2^i^	0.86	2.18	2.972 (2)	153
N3—H3···O3^i^	0.86	2.03	2.850 (2)	159
C6—H6A···O4^ii^	0.96	2.55	3.303 (3)	136
C11—H11B···O4^iii^	0.96	2.41	3.346 (3)	164

Symmetry codes: (i) *y*, −*x*+1, *z*−1/4; (ii) *x*−1, *y*, *z*; (iii) *y*, −*x*+2, *z*−1/4.
